# *R*^2^*NA*: Received Signal Strength (RSS) Ratio-Based Node Authentication for Body Area Network

**DOI:** 10.3390/s131216512

**Published:** 2013-12-02

**Authors:** Yang Wu, Kai Wang, Yongmei Sun, Yuefeng Ji

**Affiliations:** State Key Laboratory of Information Photonics and Optical Communications, Beijing University of Posts and Telecommunications, Beijing 100876, China; E-Mails: wuyang@bupt.edu.cn (Y.W.); wangkai_bupt2011@bupt.edu.cn (K.W.); jyf@bupt.edu.cn (Y.J.)

**Keywords:** authentication, body area network, sensor, RSS ratio

## Abstract

The body area network (BAN) is an emerging branch of wireless sensor networks for personalized applications. The services in BAN usually have a high requirement on security, especially for the medical diagnosis. One of the fundamental directions to ensure security in BAN is how to provide node authentication. Traditional research using cryptography relies on prior secrets shared among nodes, which leads to high resource cost. In addition, most existing non-cryptographic solutions exploit out-of-band (OOB) channels, but they need the help of additional hardware support or significant modifications to the system software. To avoid the above problems, this paper presents a proximity-based node authentication scheme, which only uses wireless modules equipped on sensors. With only one sensor and one control unit (CU) in BAN, we could detect a unique physical layer characteristic, namely, the difference between the received signal strength (RSS) measured on different devices in BAN. Through the above-mentioned particular difference, we can tell whether the sender is close enough to be legitimate. We validate our scheme through both theoretical analysis and experiments, which are conducted on the real Shimmer nodes. The results demonstrate that our proposed scheme has a good security performance.

## 1. Introduction

With the aged tendency of the global population, people pay more attention to healthcare requirements. Due to the advances of wireless communication and medical sensing technologies, many researchers focus on the applications of the body area network (BAN), which can provide individual healthcare services. Medical BANs consist of small and intelligent wireless sensors, which are worn on bodies or implanted in bodies to sample vital physiological signals and send the records to a base station for real-time analysis or remote diagnosis. When a disaster or ailment comes up, BANs could offer an emergency response. Recently, some BAN platforms have been put into the market. Fujitsu has developed the Inductively-Powered Ring Sensor [1], which works in conjunction with Fujitsu's developing sensor network system to be tied into the healthcare monitoring of the patient's application for Apple's iPhone. Shimmer [2] has designed a wearable sensor platform to monitor the electromyography (EMG), electrocardiograph (ECG) and galvanic skin response (GSR) of a subject.

Over the years, interoperable medical devices (IMDs), which could be seen as a type of sensor in BAN, have been widely used, but they are not designed with enough security. As a result, there are many reported hacking incidents of IMDs through the unsafe wireless channel [3,4]. In most cases, BAN's sensors, especially IMDs, send critical monitor data to the control unit (CU) or receive commands from the CU. Therefore, when an attacker pretends to be a legitimate sensor or CU joining the BAN successfully, it would bring a lot of risks to BAN users through reporting the wrong results or injecting false commands. Therefore, a BAN needs an effective node authentication scheme, which is an important stage to establish a BAN's initial trust and secure subsequent communications.

Though authentication is a traditional research issue in wireless networks, it is still faced with challenges in BAN. Traditionally, authentication is implemented by pre-deployment. However, if the initial key is pre-configured in hardware by manufactures, sensors from different companies may not work together. When the BAN sensors become ubiquitous, they could be cracked, and the pre-shared key would be stolen. Simultaneously, nodes in a BAN are given limited power and computation capabilities. Thus, node authentication in a BAN should avoid relying on cryptography. Secondly, if the initial key is distributed through the ways of wireless sensor networks (WSNs) [5-7], the end-users need to trust the whole distribution chain, which may involve less trustworthy users. Meanwhile, it cannot be expected that a BAN's user is an experienced person in configuration. Therefore, “plug-and-play” is another demand for node authentication in BAN. Finally, the authentication scheme should be compatible with existing systems. Since IMDs are implanted in humans' bodies, it is not easy to update their hardware. Consequently, node authentication in BAN should not bring in additional hardware.

In response to these challenges, we propose the received signal strength (RSS) ratio-based node authentication *(R*^2^*NA)* for BAN, which exploits the wireless character of avoiding pre-shared secrets. To the best of our knowledge, we are the first to bring the idea of MIMO (Multiple-Input Multiple-Output) into BAN for proximity-based node authentication without additional hardware. We regard the BAN user as a whole to be the receiver and every sensor or CU as an antenna, and the node in the authentication stage as the sender. The key insight of *R*^2^*NA* is that the difference in the RSS on different devices (sensors or CU) in the same BAN can imply whether the sender is nearby. When the sender is very close to one sensor in BAN, the RSS value on this sensor or CU would be far greater than that otherwise received.

By contrast, when the sender is far from the BAN, the distance between the sender and all the devices in the BAN can be regarded as similar ones, and the sender would not be able to cause a large difference in the RSS values. It is a reasonable assumption to consider that the proximity sender is legitimate, as BAN users could judge whether the sender is an adversary. We evaluate the effectiveness and efficiency of *R*^2^*NA* through abundant experiments. We find that *R*^2^*NA* works effectively in different scenarios, including the crowed scenario. The authentication time can be no more than 12 s. Compared with [8], our scheme has a relative advantage in computation cost.

The rest of the paper is organized as follows: Section 2 generally introduces the related work. Section 3 gives theoretical analysis and experiments on the RSS ratio in BAN. Section 4 is devoted to the basic aspects of our node authentication scheme, *R*^2^*NA.* Section 5 elaborates on experiments, result analysis and performance evaluation. In Section 6, we come to a conclusion and discuss some possible future directions.

## 2. Related Work

Traditionally, authentication researches in BANs exploit cryptography, such as [9−13], but they need high computational overhead or complex key management. A lightweight crypto-based authentication is proposed in [14]. However, it relies on prior-trust among the nodes or a trusted authority for key distribution, making its usability low in BAN. The device paring method is introduced in [15], which needs an additional out-of-band secure channel. Recently, non-cryptographic authentication techniques related to BAN have been developed, and they can be illustrated as follows:

Biometric-based authentication: Though the environment around human body is dynamic and complex, physiological signals are quite unique at a given time. Therefore, the idea using physiological signals for authentication and key generation was first presented by Cherukuri *et al.* [16]. Motivated by this initial idea, electrocardiogram (EEG), photoplethysmogram (PPG), iris, fingerprint, *etc.*, are used to provide security for BAN in [17−19]. These methods can meet the requirement of “plug-and-play”. Nevertheless, nodes in the same BAN need to measure the same physiological signal, which unavoidably leads to an additional hardware cost and different measuring errors.

Channel/location-based authentication: There has been increasing research on utilizing wireless channel properties for authentication. In [20], Patwari *et al.* use channel impulse response to build the temporal link signature for device identification, with a learning phase and an additional hardware platform. The schemes of [21,22] are based on monitoring the environmental signals to determine the proximity for device paring, but the devices need to be within half a wavelength distance of each other, which is restrictive for BAN. Temporal RSS variation lists are used to resist identity-based attack in [23], and this approach is brought into BAN by Shi *et al.* [8]. Different from [23], Shi *et al.* [8] exploits the distinct RSS variation behaviors between an on-body and an off-body communication channel to distinguish legitimate nodes from false ones. Nevertheless, Shi *et al.* [8] is not suitable for the crowded scenario, and it assumes that attackers' directional antenna cannot be directed towards the user. The authors of [24] propose a device paring scheme using different RSS to perform proximity detection.

Proximity-based authentication: Authentication schemes can be based on proximity detection. In many circumstances, the adversary cannot come close to the user's devices or cannot do so without being detected. This idea originates from [25]. Under the inspiration of [25,26] utilizes radio frequency (RF) and ultrasound to determine a device's proximity for controlling IMDs' access. Normally, it needs specialized hardware for high accuracy. In [27], RF distance bounding that fully uses the wireless channel is first designed, but multi-radio capabilities and additional hardware are needed. Some channel-based authentication schemes, such as [8,21,22,24], are also based on proximity. Obviously, the adversary cannot get close to the user without being detected in BAN. Additionally, the first lightweight BAN authentication scheme [8] is an example.

Motivated by [24], our work can be classified as both channel-based and proximity-based methods, but there are differences: (1) our scheme needs no additional hardware, while [24] requires at least two receiver antennas; (2) our scheme needs to consider other factors, such as shadowing, because of the on-body channel; (3) we focus on distinguishing whether a node is legitimate or not, while [24] focuses on device pairing. Additionally, compared to [8], our work has a better performance, which is illustrated in Section 5.

## 3. Unique Channel Characteristics of the RSS Ratio in BAN

In this section, we will introduce the fundamentals of the RSS ratio, channel models in BAN and the RSS ratio in BAN and give the experimental evidence.

### 3.1. RSS Ratio Basic

RSS is an indicator of the power of the received radio signal at receiving antennas. In wireless communications, without regard to any reflections or multipath, the power of the signal at the receiving antenna can be quantified using the free space propagation model as follows:

(1) $$P_{r}=P_{s}G_{s}G_{r}{\left(\frac{\lambda }{4\pi d}\right)}^{2}$$

where *G_s_* and *G_r_* are the gain of the sending and receiving antenna respectively, *P_s_* is the power at the surface of the sending antenna and *d* is the distance between the two antennas.

When *P_r_* is described in *dBm*, the formula is changed to:

(2) $$P_{r}[\text{dBm}]=P_{0}-20\log(\frac{d}{d_{0}})$$

where *P*_0_ is the power of the signal in *dBm* at distance *d*_0_ away from the sender.

Considering fast fading caused by multipath propagation, RSS is widely analyzed by using the log-normal shadowing model:

(3) $$P_{r}[\text{dBm}]=P_{0}-10\alpha \log(\frac{d}{d_{0}})+\chi_{\sigma }$$

where *α* is the path loss exponent, *χ_σ_* is the background noise (Gaussian distributed random variable) with zero mean and standard deviation *σ*, and the other parameters have the same meanings as those in Equations (1) and (2). According to [28], the value of path loss exponent *α* (>0) is different based on the type of environment. For example, *α* is two in free space.

The equations above have shown that RSS could be related to the distance between sender and receiver. Additionally, RSS is easy to obtain through wireless card in existing systems. Based on these two reasons, RSS is widely utilized for localization [29], distance ranging [30], *etc.* However, the uncertainty of *α* and *χ_σ_* makes accurate distance inference from RSS alone difficult, especially in a dynamic environment. In order to improve the stability and predictability, the RSS ratio is proposed combining multiple RSS values. If two antennas (antennas 1 and 2) are closely co-located, *d*_1_ and *d_2_* denote their distances to the signal sender, respectively. The difference between two RSS value is defined as the RSS ratio:

(4) $$r=RS\;S_{1}-RS\;S_{2}$$

where *RS* *S*_1_ and *RS* *S*_2_ are the RSS readings of antennas 1 and 2, respectively. Assuming *RS* *S*_1_ and *RS* *S*_2_ values follow the log-normal shadowing model, Equation (4) can be changed to:

(5) $$r=10\alpha \log(\frac{d_{2}}{d_{1}})+\chi_{\sigma 1}-\chi_{\sigma 2}$$

where the environmental unknowns, *P*_0_ and *d*_0_, in Equation (3) have been eliminated. *χ_σ_*_1_ and *χ_σ_*_2_ represent the background noise from the sender to antenna 1 and antenna 2, respectively. To remove *χ_σ_*_1_ - *χ_σ_*_2_ in Equation (5), let *RS* *S*_1_ and *RS* *S*_2_ denote the average of the sufficient number of RSS measurements. Then, the RSS ratio can be gotten as:

(6) $$r=10\alpha \log(\frac{d_{2}}{d_{1}})$$

When the sender is closed to antenna 1, *d*_1_ is very small, which makes *d*_2_/*d*_1_ large. That means, r is a large positive number in this situation. Similarly, When the sender is closed to antenna 2, *d*_2_ decreases, *d*_2_/*d*_1_ becomes very small, and r is a large negative number. When the sender is not in the proximity of either antenna, assuming *l* is the distance between two antennas, the largest value of |*r*| that the sender can get is (*d*_2_ + *l*)/*d*_1_. If *d*_1_ is sufficiently larger than *l*, |*r*| cannot be large. That means that a faraway sender is unable to obtain a large RSS ratio.

## 3.2. Channel Model in Body Area Network

As sensors' positions are on or inside the body, the BAN channel models need to consider the impact of the human body and activities. In [31], IEEE 802.15.6 group defines three types of nodes as follows:

(1) Implant node: a node that is placed inside the human body.
(2) Body surface node: a node that is placed on the surface of human skin.
(3) External node: a node that is not in contact with human skin.

Based on the different node types, Yazdandoost *et al.* [31] gives four channel models (CMs) shown in Table 1. In this paper, with the hardware constraints, we focus on the latter two channel models (on-body and off-body). By detecting the RSS ratio, we can tell whether the channel between the sender and BAN is on-body or off-body to authenticate the sender. By the way, according to [31], when body is in stationary or slow motion state, sensors within the range of 0.4 m from the body communicate through the on-body channel. However, in real life, it may be smaller and more dynamic.

### 3.3. RSS Ratio in Body Area Network

Due to the variation in the environment surrounding the body or even the movement of the body parts, the power of the signal at the receiving sensor will be different from the mean value for a given distance, as shown in Equation (3). This phenomenon is called shadowing. Considering the shadowing in BAN, the stationary and non-stationary position of body should be taken into account.

When considering shadowing, the power of the signal at the receiving sensor can be expressed by:

(7) $$P_{r}[\text{dBm}]=P_{0}-10\alpha \log(\frac{d}{d_{0}})+\chi_{\sigma }+S$$

where *S* represents the shadowing component.

**Figure 1.**
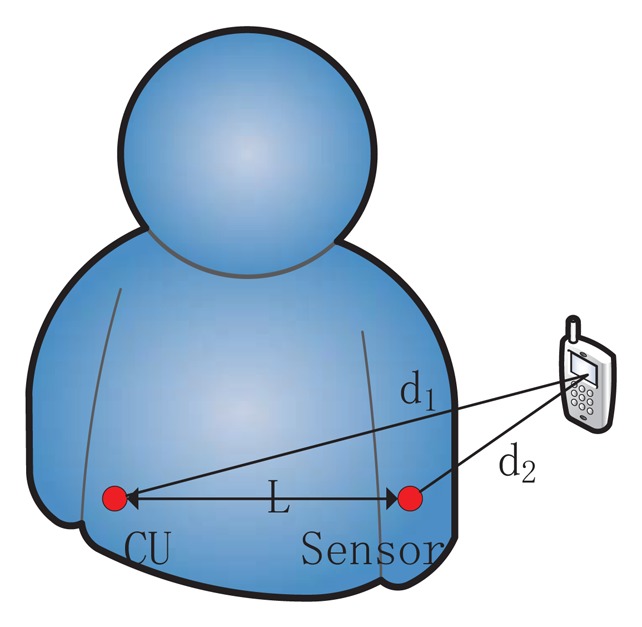
Illustration of the setting for received signal strength (RSS) ratio in BAN.

To describe the RSS ratio in BAN, we assume the setting shown in Figure 1. Then, the RSS ratio in BAN is changed to:

(8) $$r=10\alpha \log(\frac{d_{2}}{d_{1}})+S_{1}-S_{2}$$

where *S*_1_ and *S*_2_ are the shadowing component from the sender to the CU and sensor, respectively. Since *S*_1_ – *S_2_* ranges from −∞ to +∞, we convert it to 10*α* log *K*, in which *α* is the same with that in Equation (8). Thus, *S*_1_ – *S*_2_ can be expressed as a function of *K*, and *K* is impacted by some factors by the nodes' position, body type, human motion, *etc*. Above all, Equation (8) could be represented as follows:

(9) $$r=10\alpha \log(K\frac{d_{2}}{d_{1}})$$

In the situation of Figure 1, it can be deduced that CU's shadowing component is larger than the sensor's, as the signal travels more through the body to the CU than to sensor. That means *S*_1_ – *S*_2_< 0 and *K* < 1. At the same time, *d*_2_ is less than *d*_1_, so *r* is a larger negative number with shadowing in BAN than that mentioned in Section 3.1. Likewise, if the sender is close to CU, we will obtain *S*_1_ – *S*_2_ > 0, *K* > 1 and *d*_2_/*d*_1_ > 1. As a result, *r* is a larger positive number with shadowing in BAN than that mentioned in Section 3.1. When the sender is far away, the shadowing component, *S*, cannot play a part, and Equation (6) would work. Based on this, we can choose appropriate thresholds *r_h_* (when the sender is close to CU) and *r_l_* (when the sender is close to sensor) to distinguish a faraway attacker from a legitimate nearby sensor.

### 3.4. Experimental Evidences

**Figure 2.**
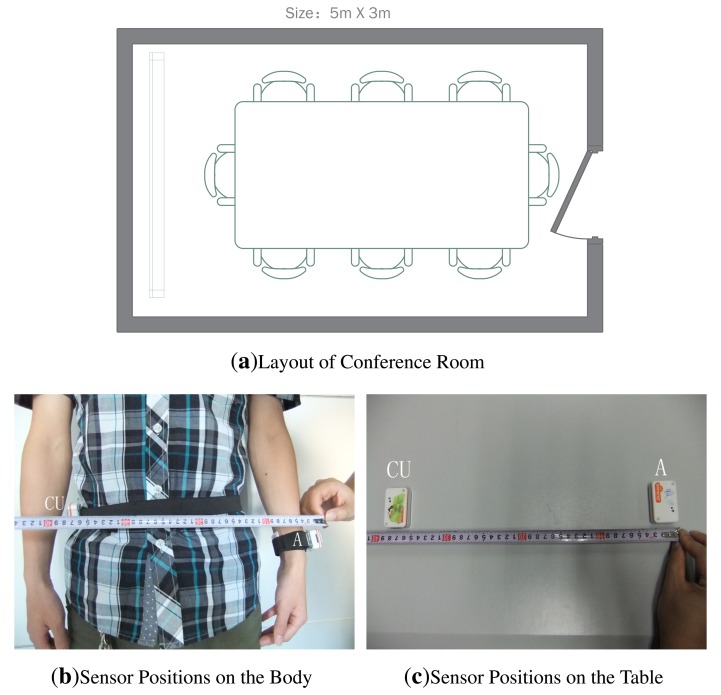
Experimental layout for indoor environment and sensor placement.

To testify the effect of human body on the RSS ratio, we carry out experiments using Shimmer sensors, which are running TinyOS and operating in the 2.4 GHz band. With logarithmic units, their radio antennas (CC2420) can measure signal power and output RSS value. Our experiments are conducted under a semi-controlled scenario, a conference room as shown in Figure 2a, with only the researchers present. For scenario 1, in order to take account of the effect of human body, one person wears one sensor (CU) on the right waist and another one (A) on the left wrist, as shown in Figure 2b. For scenario 2, we just put CU and A on the table, as shown in Figure 2c. To distinguish on-body and off-body channels, the experiments are done by putting the sender at different distances from the sensors. Moreover, two different signal-sending directions are considered. The details of our setup are listed in Table 2.

**Figure 3.**
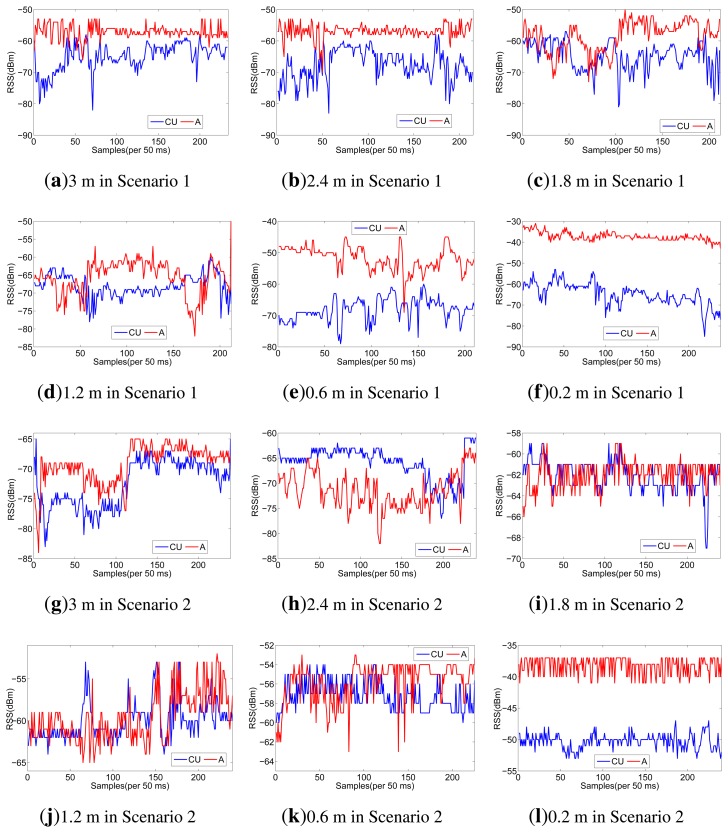
RSS traces when the sender forwards along the Line of A and CU.

**Figure 4.**
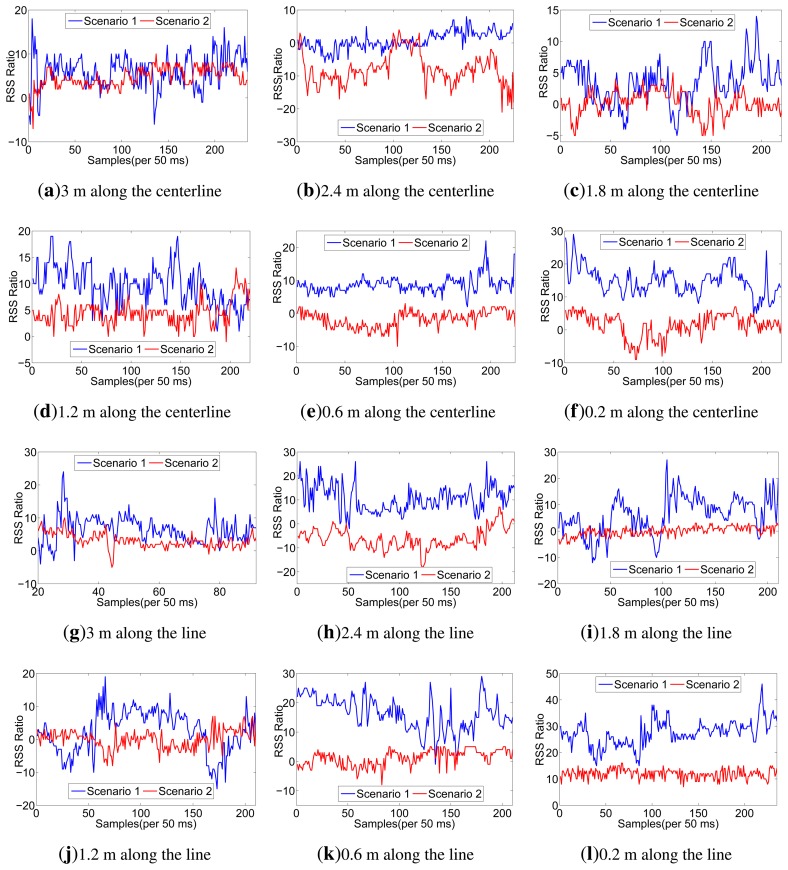
RSS ratio (A-CU) traces in all experiments.

Considering the space limitations of this paper, we only list RSS traces when the sender moves along the line of A and CU in Figure 3, but all the RSS ratio (A-CU) traces are listed in Figure 4. As shown in Figure 3, in scenario 1, RSS traces within 0.6 m have less fluctuations than RSS traces in scenario 2. Even in scenario 1 itself, RSS traces within 0.6 m have less fluctuations than other traces. It can be concluded that body shadowing leads to the ineffectiveness of the multipath effect, which becomes increasingly obvious from 0.6 m to 0.2 m, as shown in Figure 3f. Though we cannot tell whether there is an on-body channel at 0.6 m, we can safely conclude that the on-body channel works at 0.2 m. From Figure 4, we can find that the RSS ratio is bigger in scenario 1 than that in scenario 2 in most cases. The experiments verify our inference in Section 3.3. The human body would enlargen the RSS ratio, and the RSS ratio could be used to distinguished on-body channels and off-body channels. We still need to consider some other factors, such as the distance between two sensors (deployed position) and body type, which would be further explored in Section 5.

## 4. Design

In this section, we will propose the attack model, our design goal, the basic design and some discussions.

### 4.1. Attack Model

Impersonation attacks is mainly considered in this paper. We wish to ensure no faraway attacker can act as a legitimate node to join the BAN. We assume the attackers are powerful: the attackers may own advanced hardware; they can have arbitrarily high transmission power and adjust the transmission power arbitrarily; they can forge physical addresses, like Media Access Control (MAC) address, and replay or inject false data; they can sniff all the traffic of the BAN. In addition, the attacker may have knowledge about the wireless environment around the BAN. For example, they could survey the location where the BAN will be set up by measuring the channel in advance and can derive corresponding signal propagation models. The attackers can also know security schemes, the transmission technology and the technical specs of the sensors and CU. However, we exclude compromising a legitimate device (CU or sensors in BAN) and jamming the wireless channel.

### 4.2. Design Goal

Our goal is to build a practical, reliable scheme for node authentication in BAN. We only consider one-way authentication: only BAN authenticates the sender. In many applications, if BAN needs to send data to a server, it will normally log on to the Internet to be connected to a remote server via computers or smart phones instead of BAN's own wireless communication, and this log-on process is an authentication that needs not to be considered in this paper. A particular example is remote healthcare: BAN sensors measure and send physical signals, which the doctor will get through the Internet or via using a device. The former Internet scenario can make use of the internet authentication, while the BAN needs to authenticate the device in the latter scenario. Therefore, one-way authentication is enough. After authentication, the sender can share a secret key with the CU to protect the communication between them. We do not refer to key distribution, for a lot of works, such as [32-34], have been done.

Besides, the following properties should be contained in the authentication scheme for BAN:

(1) Speed: latency may cause a very different consequence in emergency.
(2) Efficiency: due to the resource limitations of sensors in BAN, resource consumption must be minimized.
(3) Compatibility: as mentioned before, it is hard to update IMDs' hardware, so the security scheme should depend on off-the-shelf hardware and should not make big changes to the existing system.
(4) Usability: since the BAN users cannot be expected to be experts, “plug-and-play” is needed.
(5) Applicability: the security scheme should work under as many types of scenarios as possible.

### 4.3. Basic Scheme

Before getting to the *R*^2^*NA* protocol, we first acknowledge that the model of the body area network is as follows: *n* (*n* ⩾ 1) sensors are carried on the body of a man to measure and collect physiological data about the user (e.g., heart rate, electromyography, glucose level, *etc.*). The sensors send the data to a CU, which is in one-hop range of the sensors. The sensors are resource constrained, but the CU could be a more powerful device, such as a smart phone or Personal Digital Assistant (PDA). The CU processes or aggregates the data, then stores and waits to send them to remote/locate legitimate users. All the devices in a BAN have a wireless module, which enables them to communicate over wireless channel (e.g., ZigBee, Bluetooth, WiFi, *etc.*). No additional hardware, such as multiple antennas, is assumed, neither is an out-of-band communication channel.

*R*^2^*NA* exploits the RSS ratio mentioned in Section 3. To get different RSS readings for the RSS ratio, the receiver needs to own two antennas. Since a BAN is composed of at least one sensor and one CU, each device owns an antenna. Therefore, we regard the BAN as a whole, and two antennas can be satisfied. However, another problem comes out: how do we make the CU know the two RSS readings? By communication. Then, how does one avoid interference with the sender? We introduce time division.

Step 1: When the CU receives a request to join the BAN, the CU broadcasts a hello message *M =* (*x*, *t*_0_, *t*) to nearby devices. The meaning of the hello message is asking all the responding devices (legitimate sensors and the requester) to send back acknowledgment of every *t* ms after *x* s and continue for *t*_0_ s. To avoid the attacker measuring the real-time channel between itself and the CU, the CU will not respond during the *t*_0_ s.

Step 2: Once receiving the hello message, each of the responding devices generates a random number, *t_r_*, calculates *t_i_* = *t_r_* mod *t* and then sends *t_i_* back to the CU. After all the random numbers (*t_i_*s) are collected, the CU compares each to make sure there are no identical ones to avoid communication collisions in the future. If duplications are found, the CU will return step 1 to send a new hello message. All of Step 2 must be done in *x* s.

Step 3: After all the random numbers are confirmed by CU, the respond devices will repeatedly send messages to the CU after *ϰ* s plus *t_i_* ms. The number of packets, *NT*, is 1,000 × *t*_0_/*t.* However, there are differences between the requester and legitimate sensors: the former only sends empty message for the latter and the CU to measure the RSS value, while the messages sent by the latter contain their previous RSS readings. At last CU would get all RSS readings to calculate the RSS ratio, *r.*

The system parameters, *x, t*_0_ and *t*, can be set appropriately. For *x*, it must be large enough to finish Step 2, but a big value will increase latency. For *t*_0_ and *t*, they should be set to make *NT* large enough to remove the noise impact (*χ_σ_* in Equation (7)), and latency should also be considered.

Step 4: Having a sufficient number of consecutive *r* values, the mean of them can be gotten by CU. If the mean is greater than a positive *r_h_* or less than a negative threshold, *r_l_*, the requester is allowed to join the BAN.

### 4.4. Discussion

Due to human motion and social activities, BAN may be in a crowded scene, such as on public transportation, or encounters the situation that someone just walks by the user. All that is described above means that the attacker may be very close to the BAN user. However, since the devices of BAN are scattered on the body, it would be very difficult for the attacker to place his sender close to more than two devices in BAN sequentially without being inconspicuous. Therefore, our scheme requires the sender to be placed close to the CU and one legitimate sensor (for a higher security level, the sender can be asked to be placed close to more legitimate sensors).

**Figure 5.**
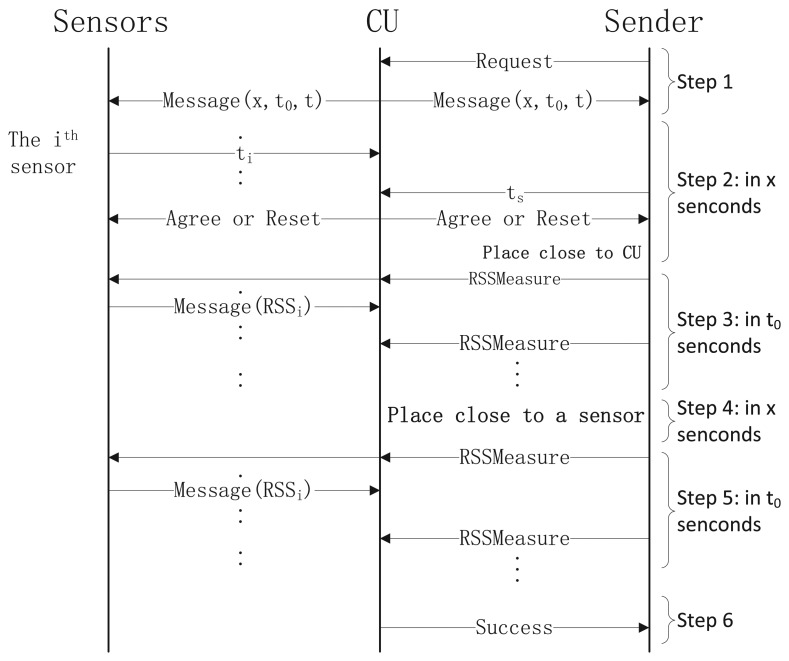
The final protocol.

Based on the observations above, the final protocol is presented in Figure 5. Steps 1-3 are the same as those in Section 4.3. The purpose of Steps 4 and 5 is to reduce the probability of attacks in the crowded scenarios, such as “walk-by”. In Step 6, CU examines all the RSS values and calculates the RSS ratio. If CU detects a sufficient number of consecutive packets whose mean of *r* is above a threshold, *r_h_*, then CU knows that the requester is nearby him. Similarly, the CU detects if the sender is then nearby a sensor. By satisfying these conditions, the CU sends a success message to the requester.

## 5. Experiments

In this section, we will first present our experimental setup and results. Specifically, the following factors are taken into account: The placement of the body sensor, body type, human motion and the location of the attacker. Then, we will evaluate the security and efficiency of our scheme.

### 5.1. Setup

**Figure 6.**
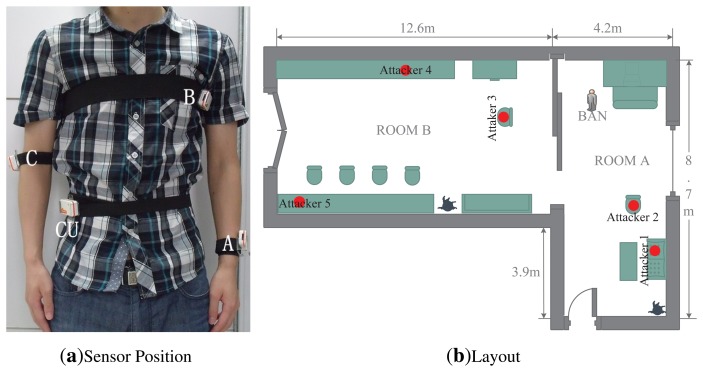
Sensor placement on the human body and layout for experiments.

Our experiments still use Shimmer sensors. Considering the position of the sensors, we configure four Shimmer sensors worn on the right waist, left chest, right arm and left wrist, respectively. As shown in Figure 6a, the sensor located on the right waist (CU) emulates the controller for data collection and aggregation, and other sensors are labeled from A to C. Our experiments are conducted in a typical indoor environment, and its layout is depicted in Figure 6b. The size of room A is 8.7 m (length) × 4.2 m (width) × 2.4 m (height). The size of room B is 12.6 m (length) × 4.8 m (width) × 2.4 m (height). Room A and B are connected through a sliding door. We also put several Shimmer sensors (numbered from 1 to 5) at different locations to simulate attackers. Considering the differences among individuals, four persons participate in our experiments, and their demographic data are shown in Table 3.

### 5.2. Prototype

Our experiments are divided into several plans. Plans 1 and 2 are to confirm some impacts of other factors, and the other plans are to validate our proposed scheme in different scenarios.

Plans 1, 2: The experiments are conducted on users 1−3 in room A. The BAN user stands in room A, as shown in Figure 6b. All the attackers are not deployed. For Plan 1, a sender is placed 3.5 m away from the CU, facing the user. For Plan 2, a sender is placed at the side of the user and also 3.5 m away from A. In both of the plans, the sender sends a packet every 50 ms, and the number of packets is about 140. We repeated this measurements for different distances: 3 m, 2.5 m, 2 m, 1.5 m, 1 m, 0.8 m, 0.6 m, 0.4 m, 0.2 m and 0.1 m.

**Figure 7.**
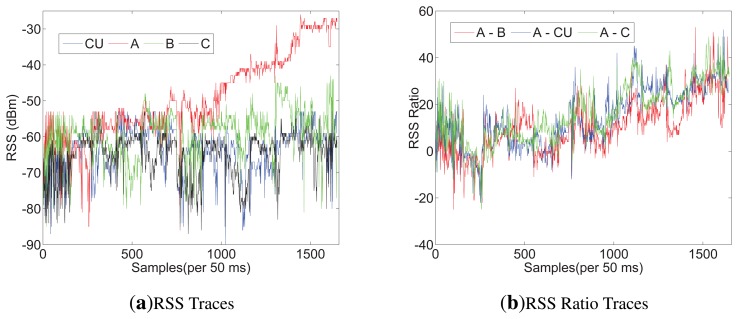
The RSS and RSS ratio of user 2 measured in Plan 2.

**Figure 8.**
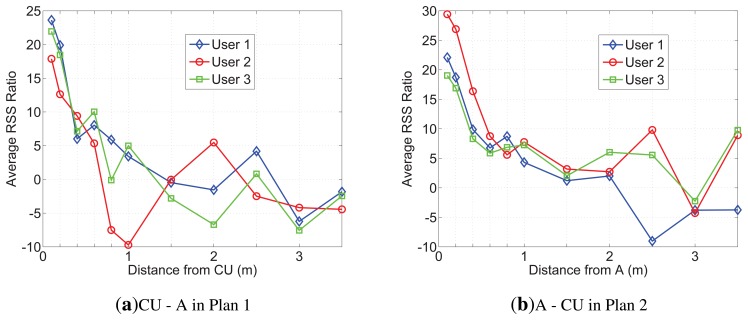
The average RSS ratio of CU-A in Plan 1 and A-CU in Plan 2.

Because of limited space, only the RSS and RSS ratio of user 2 measured in Plan 2 are presented in Figure 7. From Figure 7a, we can find that A's RSS trace increases more than others' after 1,000 samples, obviously, which indicates in 0.8m, the average RSS ratio would enlargen. Additionally, after 1,400 samples (about in 0.2 m), As RSS trace is more stable than before. That can be explained by the reason that the direct path (DP) is the dominant path among all the multipath components on the on-body channel. Figure 7b shows that after 1,000 samples (in 0.8 m), the RSS ratio between A and CU is the same stable as that between A and C and is more stable than that between A and B. We list the average RSS ratio, |*r*|, of Plans 1 and 2 on the on-body channel in Table 4. From Table 4, we can see that *CU* − *A* and *A* − *C* are the biggest; *A* − *C* is greater than *CU* − *A* in every row. The reason could be concluded as follows: (1) |*r*| rises with the increase of distance between two receiving sensors; (2) shadowing would bring parameter *K* into Equation (9), and *K* is determined by the distance between two receiving sensors and the body type. In BAN, the distance between two sensors relies on the position of deployment. Our scheme expects more shadowing and a long distance, while other applications call for less shadowing and a short distance to have better communications. Additionally, there are some physical signs that need to deploy sensors at the specific position. Considering the combining factors, we suggest that A is suitable for efficiency with the CU tied on the other side of the waist. All the average RSS ratios between CU and A in Plans 1 and 2 are shown in Figure 8. Based on our measurements, it is hard for the sender to make the average RSS ratio, |*r*|, more than 10 through the off-body channel. Therefore, we set *r_h_* > 12 and *r_l_* < −12 for A and CU. To validate our scheme in different scenarios, the following plans only deploy CU and A on the users, and the parameters, *x*, *t*_0_ and *t*, are set to be 1, 5 and 50, respectively.

Plan 3, 4: For Plan 3, the experiments are conducted on users 2 and 3 in room A. The BAN user sits beside attacker 1 in room A, doing social activities with other persons to simulate the crowded scenario. Attackers 1 and 2 are about 0.8 m and 2 m away from the user, respectively. Attackers 3−5 are not deployed. For plan 4, the experiments are conducted on users 1 and 4 in room B. The user randomly walks in the room. There are also several persons working in the room. In this plan, attackers 3−5 are deployed. In both plans, all the attackers send request messages every 250 ms, but CU only responds one at a time.

The results of Plans 3 and 4 are listed in Table 5. Since attackers and BAN users do not change their position in Plan 3, attackers can only make a positive RSS ratio. In Plan 4, users walk randomly, which gives attackers a chance to produce a negative RSS ratio. However, it is hard to produce a big positive number greater than *r_h_* or negative number less than *r_l_*. Furthermore, even though the attacker is put near the users (attacker 1 in Plan 3), it is unable to get close to another sensor.

### 5.3. Security Evaluation

We evaluate the security of our scheme through authenticating a sender at different distances for different channels from the users in Plans 3 and 4:

(1) Close-range: The sender is placed no more than 0.2 m away from one sensor on the body. In this situation, the channels between the sender and all sensors must be the on-body channel.
(2) Mid-range: The sender is between 0.2 m-0.6 m away from one sensor. The channels between the sender and all sensors cannot be distinguished.
(3) Long-range: The sender is more than 0.6 m away from one sensor. The channels between the sender and all sensors must be the off-body channel.

For each distance, the sender is put along the line of CU and A. For each test, we made 30 attempts. Table 6 shows that the success rates for close-range authentication in both plans are nearly 100%. The failed authentication in this range may have happened when the user moves the upper part of the body significantly By contrast, our scheme rejects all the attempts in the long range. In the mid-range, we also reject most of the attempts, and we suppose that it is caused by human random activity. From the results, we believe that our scheme provides excellent security and works effectively in different scenarios.

### 5.4. Security against Attacks

A possible method to attack *R*^2^*NA* is using the multipath effect. RSS can become stronger or weaker if there is a constructive or destructive superposition of the signals coming from different paths. The multipath effect is more obvious in an indoor environment than in an outside environment, because the surface of the floor, ceiling, walls, furniture and even people can reflect the wireless signal. Using our scheme, when the sender is close to the BAN user, the multipath effect will unlikely affect the RSS values significantly. However, if a distant attacker knows the channel models of the environment, he could take advantage of the multipath effect to cause a large RSS ratio to break our scheme. However, this attack can be mitigated by adding frequency hopping into our scheme. With frequency hopping, it is very difficult for the attacker to find a path length that keeps the RSS ratio high in all the channels, because optimal path length for the attacker in different channels is quite different. It is straightforward to add frequency hopping in our scheme: the sender sends *RSSMeasure* packets, while cycling through all the channels instead of using only one channel. Therefore, it is difficult for attackers to use the multipath effect in practice.

Additionally, in most cases of real life, the user of the BAN moves randomly, making the attacker hard to measure by the channel models of the environment. Moreover, the attacker cannot even get an accurate estimation on the channel state information (CSI) based on its observation of the reverse channel (the channel from the BAN nodes to the attacker), because of the following two reasons. First, our scheme does not require the BAN nodes to send messages at the same time. Therefore, the attacker cannot measure the reverse CSI of both channels. Second, even if the BAN nodes send signals at the same time, the CSI of the reverse channel may be different from that of the forward one, because reciprocity may not hold, due to non-symmetric noise.

Beam-forming attack could also be attempted by a powerful distant attacker to form special beams to cause a large difference between the RSS values at the CU and sensors. In practice, however, this attack would be very difficult, if not impossible; the beam forming attacker would need a narrow-width main lobe. The lobe width is inversely proportional to the size of the antenna arrays. Since the distance between the CU and sensors is usually small (typically less than one meter), the attacker would need a very large antenna array, which, in many, situations would raise suspicion. When the attacker is far from an indoor receiver, the multipath effect would likely distort the intended beam too much. According to the reasons mentioned before, attacker cannot measure the channel models to mitigate the multipath effect.

### 5.5. Performance Evaluation

The performance can be evaluated by speed (authentication time), efficiency (computation and communication costs), compatibility, usability and applicability, which are the performance metrics we use to compare our method with BANA (the previous lightweight body area network authentication scheme in [8]), and a classic authentication scheme based on RSA.

Speed (authentication time): Our scheme consists of six steps, and the time costs of step 2 to 5 is fixed. As to step 1 and 6, they only need milliseconds (time cost of sensor response), so their time cost can be neglected. Since the parameters *x*, *t*_0_ and *t* are set to be 1, 5 and 50 respectively, the authentication time of our scheme is 12 s. We could also decrease our authentication time by resetting *t*_0_ and *t*. To ensure sensors receive enough samples for analysis, *NT* should be kept no less than 100. But we could decrease the time interval *t*, which only needs to be greater than time cost of sensor response. If *t* is set to be 20, *t*_0_ could be reduced to 2 s. Then, the time cost of our scheme would be 6 s. In [8], their authentication time reaches 12 s in most cases. For RSA scheme, we set 1,024-bit public keys for certificate. Running on the shimmer sensor, RSA takes nearly 80 s to do authentication. So our scheme and BANA have similar performances on this metric, but both of them are better than RSA scheme.

Efficiency (computation and communication costs): The computation cost for each body sensor, such as A, is the same as BANA's, since both of the two schemes have no time-consuming task executed on the sensor. On the CU's side, BANA needs to run some clustering algorithm, whose complexity can be at least *O*(*n*^3^ log *n*), where n corresponds to the number of sensor nodes, which is a relatively small number. The computation complexity of the RSA scheme is *O*(ln *m*l*n*^2^*n*+ln^2^*m*), where n is the times of modular multiplication and m is the times of modular exponentiation. Moreover, the value of them (m and n) should be great. However, our scheme only requires calculating the average of the RSS ratio and comparing them, whose complexity is *O*(*k*) (here, k is constant). Obviously, our computation cost is less than BANA's, and both of their computation costs are less than that of the RSA scheme. The communication cost of our scheme for each body sensor is mainly caused by sending messages to the CU every *t* for reporting the RSS value; so, the communication cost of our scheme is the same as that of BANA. According to [8], though the RSA scheme needs less communications, its computational cost is much higher than that of BANA, making the total cost of RSA higher than BANA's. Thus, in these three schemes, our proposed one has the best performance on this metric.

Compatibility: The three security schemes all depend on off-the-shelf hardware, which means they can be easily used by updating software. These three schemes have the same performance in terms of compatibility.

Usability: Our scheme and BANA do not need configuration before the authentication process. They are therefore easily utilized by inexperienced users. However, the RSA scheme needs further configuration, which may become problematic for untrained users. Therefore, our scheme and BANA are the same in this respect, and they are better than the RSA scheme.

Applicability: Since the RSA scheme is not only for BAN, our scheme is just compared with BANA on this metric. Our scheme has considered attacks in the crowded scenarios, to which BANA does not refer. Therefore, our scheme is better than BANA with respect to applicability.

## 6. Conclusions

This paper proposes a lightweight proximity-based authentication scheme, *R*^2^*NA*, for body area networks. The scheme takes advantage of a characteristic of wireless channels without additional hardware. When a sender is close enough to a CU or one sensor in a BAN, the CU can observe a large difference between the power measured on different devices, whereas a faraway sender would be unable to induce this large difference. We validate our scheme through theoretical analysis and experimental measurements. We discussed factors that may affect our scheme, including sensor position, human motion, the environment and body type. Finally, we evaluated the security and performance of our scheme. The experiment shows that our scheme meets the requirements of BAN authentication.

Our success authentication rate is nearly 100% when the sender is no more than 0.2 m away from one legitimate device in a BAN. Additionally, our scheme works effectively even in the crowded scenario. Furthermore, we have noted that a work of research [35] uses three antennas to calculate the RSS ratio for more stable and predictable analysis, which we would explore in future work.

## Figures and Tables

**Table 1.** Channel models in body area network (BAN). CM, channel model.

**Description**	**Channel Model**
Implant to Implant	CM1 (In-Body)
Implant to Body Surface or External	CM2 (In-Body)
Body Surface to Body Surface	CM3(On-Body)
Body Surface to External	CM4(Off-Body)

**Table 2.** Experimental setup.

	**Scenario 1**	**Scenario 2**
Position of sensor A	Left wrist	On the table
Position of CU	Right waist	On the table
Distance between A and CU	37 cm	
Distance between A (CU) and sender	3 m, 2.4 m, 1.8 m, 1.2 m, 0.6 m, 0.2 m	
Forward direction of the sender	Along the line of A and CU, along the centerline of A and CU	
Channel description	On-body, Off-body	-
Transmission interval	50 ms	

**Table 3.** Details of the test user.

	**Gender**	**Height (cm)**	**Weight (kg)**
User 1	Male	175	80
User 2	Male	172	60
User 3	Male	180	62
User 4	Female	158	45

**Table 4.** Part results of Plans 1 and 2. Distance in parentheses means how far the sender is to the user.

**Average RSS Ratio**	**Plan 1**			**Plan 2**		

	**CU-A**	**CUB**	**CU-C**	**A-CU**	**A-B**	**A-C**
User l (0.1m)	23.63	14.75	15.27	22.10	16.07	27.28
User 1 (0.2 m)	19.9	13.73	13.86	18.72	14.44	20.62
User 2 (0.1m)	17.88	17.09	15.46	29.42	28.91	33.44
User 2 (0.2 m)	12.62	11.51	10.85	26.89	17.28	25.33
User 3 (0.1m)	21.95	15.78	17.55	19.04	13.06	24.14
User 3 (0.2 m)	18.46	12.13	15.37	16.89	10.81	19.58

**Table 5.** Results of Plans 3 and 4.

**Maximum Average RSS Ratio**	**Plan 3**		**Plan 4**	

	**User 2**	**User 3**	**User 1**	**User 4**
Attacker 1	13.60	9.76	**-**	**-**
Attacker 2	2.73	1.73	**-**	**-**
Attacker 3	**-**	**-**	7.37, −7.68	7.42, −7.86
Attacker 4	**-**	**-**	7.58,−8.15	7.13,−7.81
Attacker 5	**-**	**-**	6.59, -8.38	6.39,-7.11

**Table 6.** Authentication accuracy.

	**Success Rate**		

	**< 0.2 m On-Body**	**(0.2 m, 0.6 m)**	**>0.6m Off-Body**
User 1	100%	6.7%	0%
User 2	100%	10%	0%
User 3	96.7%	3.3%	0%
User 4	100%	0%	0%
Plan 3(sitting)	98.3%	3.3%	0%
Plan 4(walking)	100%	6.7%	0%
